# Chronic Alcohol Exposure Renders Epithelial Cells Vulnerable to Bacterial Infection

**DOI:** 10.1371/journal.pone.0054646

**Published:** 2013-01-24

**Authors:** Stephen Wood, Ravi Pithadia, Tooba Rehman, Lijuan Zhang, Jennifer Plichta, Katherine A. Radek, Christopher Forsyth, Ali Keshavarzian, Sasha H. Shafikhani

**Affiliations:** 1 Department of Immunology/Microbiology, Rush University Medical Center, Chicago, Illinois, United States of America; 2 Department of Surgery, Burn and Shock Trauma Institute, Loyola University Chicago, Health Sciences Campus, Maywood, Illinois, United States of America; 3 Department of Internal Medicine, Rush University Medical Center, Chicago, Illinois, United States of America; University of Chicago, United States of America

## Abstract

Despite two centuries of reports linking alcohol consumption with enhanced susceptibility to bacterial infections and in particular gut-derived bacteria, there have been no studies or model systems to assess the impact of long-term alcohol exposure on the ability of the epithelial barrier to withstand bacterial infection. It is well established that acute alcohol exposure leads to reduction in tight and adherens junctions, which in turn leads to increases in epithelial cellular permeability to bacterial products, leading to endotoxemia and a variety of deleterious effects in both rodents and human. We hypothesized that reduced fortification at junctional structures should also reduce the epithelial barrier’s capacity to maintain its integrity in the face of bacterial challenge thus rendering epithelial cells more vulnerable to infection. In this study, we established a cell-culture based model system for long-term alcohol exposure to assess the impact of chronic alcohol exposure on the ability of Caco-2 intestinal epithelial cells to withstand infection when facing pathogenic bacteria under the intact or wounded conditions. We report that daily treatment with 0.2% ethanol for two months rendered Caco-2 cells far more susceptible to wound damage and cytotoxicity caused by most but not all bacterial pathogens tested in our studies. Consistent with acute alcohol exposure, long-term ethanol exposure also adversely impacted tight junction structures, but in contrast, it did not affect the adherens junction. Finally, alcohol-treated cells partially regained their ability to withstand infection when ethanol treatment was ceased for two weeks, indicating that alcohol’s deleterious effects on cells may be reversible.

## Introduction

It is generally accepted that exposure to 0.2% (v/v) or higher ethanol concentrations (up to 2%) causes intestinal barrier dysfunction by disrupting tight and adherens junctional structures [1], [2], [3], resulting in increased epithelial permeability, which allows bacterial products to passively cross the barrier, culminating in endotoxemia-induced tissue and organ damage [1], [4], [5], [6], [7], [8], [9]. One thing that has not received proper scrutiny is the impact of long-term alcohol exposure on bacterial infection.

The link between alcohol consumption and bacterial infection dates back to two centuries ago when Benjamin Rush listed tuberculosis, pneumonia, and yellow fever as complications of alcoholism [10]. Chronic alcohol consumption has been linked to higher incidence of bacterial pneumonia [11], [12]. Alcohol consumption before burn wounds has been shown to increase the risk of pulmonary infection with *Pseudomonas aeruginosa* [13]. Alcoholics have been shown to be at a greater risk for travelers’ diarrhea and for infections with *Klebsiella pneumoniae*, *Haemophilus influenzae*, and *Streptococcus pneumoniae* [14], [15], [16], [17], [18]. The rarely occurring *Bartonella quintana* bacteremia which usually occur in HIV patients, has also been reported in chronic alcoholics [19]. To date however, there have been no studies to examine the impact of long-term alcohol exposure on the ability of epithelial cells’ to maintain barrier integrity in the face of bacterial onslaught particularly in places like the gut when they are in constant battle.

We established a cell culture-based alcohol model system to assess the impact of long-term alcohol exposure on the ability of Caco-2 epithelial cells to withstand bacterial infection under the intact (uninjured) and wounded conditions. We report here that daily exposure to 0.2% ethanol (EtOH) for two months rendered Caco-2 cells substantially more vulnerable to cellular damage caused by most but not all bacterial pathogens both in the intact and in the wound models. Consistent with acute alcohol exposure, we found that long-term exposure to alcohol weakens the tight junction structures. In contrast to acute alcohol exposure, however, long-term exposure to alcohol had no effect on adherens junction. Interestingly, stoppage of alcohol treatment for two weeks led to a partial recovery phenotype in the alcohol-treated Caco-2 cells, suggesting that alcohol’s deleterious effects may be reversible.

## Results

### Establishing a Long-term Alcohol Exposure Model

For these studies, we chose Caco-2 intestinal epithelial cell because of its origin and direct link to the gut; because of alcoholic liver disease and the alcohol-gut-liver axis; and because it has been the choice cell type for previous studies which have examined the impact of acute alcohol exposure on epithelial cell physiology [1], [4], [5], [6], [7], [8], [9], [20].

**Figure 1:**
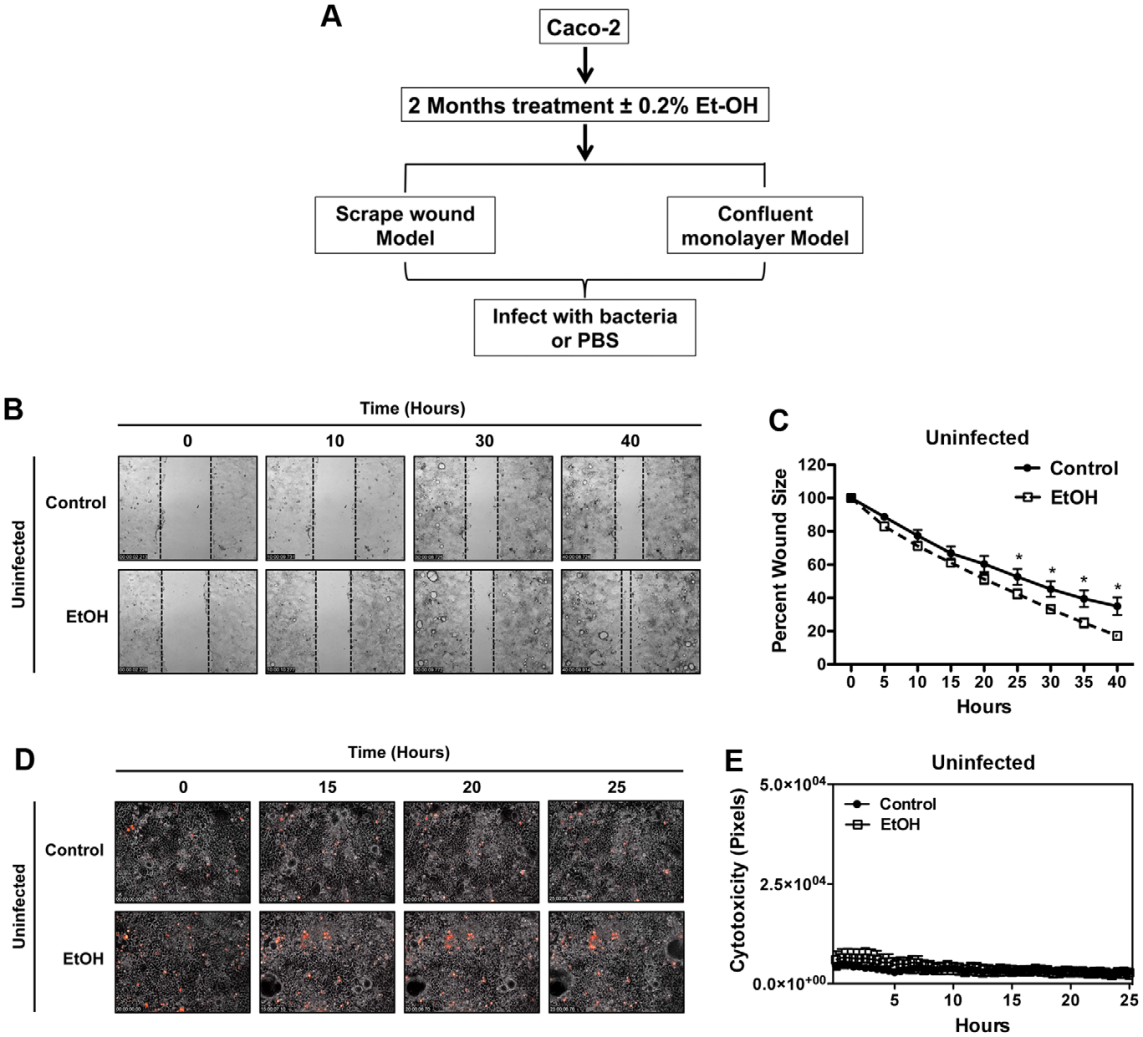
Establishing the long-term alcohol model. (**A**) A schematic diagram depicting the long-term alcohol exposure model used in these studies is shown**.** Caco-2 cells were treated with 0.2% ethanol or 0.2% H_2_O (control) daily for 2 months. Confluent monolayers were either kept intact (uninjured) or scrape-wounded as described in materials and methods (Wound model). (**B**) Control and EtOH Caco-2 confluent monolayers were scrape wounded. The impact of long-term ethanol exposure on the healing dynamics of Caco-2 wounded epithelium was measured by time-lapse videomicroscopy. Representative frames at indicated time points post wounding are shown. (**C**) Wound areas were measured and the tabulated results relative to the original wound size, indicating that EtOH treated cells heal faster than the control. (**D & E**) The cytotoxic impact of long-term alcohol exposure on Caco-2 confluent monolayer was evaluated by IF time-lapse videomicroscopy, using PI uptake (red cells are dead) as described in materials and methods. Representative frames at indicated time points are shown in (D) and the results were tabulated from each frame and are shown in (E), indicating that EtOH treatment does not render Caco-2 cells prone to enhanced cytotoxicity. Data were plotted as the Mean ± SEM (* indicates significance with *p*<0.05, n = 3). Arrows point to cluster of cells moving inward.

Our strategy to generate a long-term alcohol exposure model is depicted in Fig. 1A and described in Materials and Methods. Briefly, adherent Caco-2 cells received fresh medium containing either 0.2% (v/v) ethanol (EtOH) or 0.2% H_2_O (Control) once daily for 60 days. During this period, cells were passaged approximately every 3 days when they reached about 80% confluency. The EtOH or control Caco-2 confluent monolayers were either scratch wounded as described [21], [22] to represent the wound condition, or kept uninjured and used as the model for intact monolayer. Various pathogenic bacteria (Table S1) were then added at a multiplicity of infection (M.O.I) of approximately 5 and their impact on junctional structures, wound healing and cytotoxicity were analyzed with static or live Immunofluorescent microscopy as described in materials and methods section and [21], [23], [24].

Interestingly, in the absence of bacterial infection, alcohol-treated Caco-2 cells healed significantly faster compared to control cells (Figs. 1B and 1C and Movie S1). Also of note, alcohol-treated Caco-2 cells exhibited slightly higher levels of cytotoxicity as compared to control, particularly during early time points (Figs. 1D and 1E and Movie S2).

### Long-term Alcohol Exposure Reduces the Epithelial Barrier Capacity to Withstand Bacterial Infection

**Figure 2:**
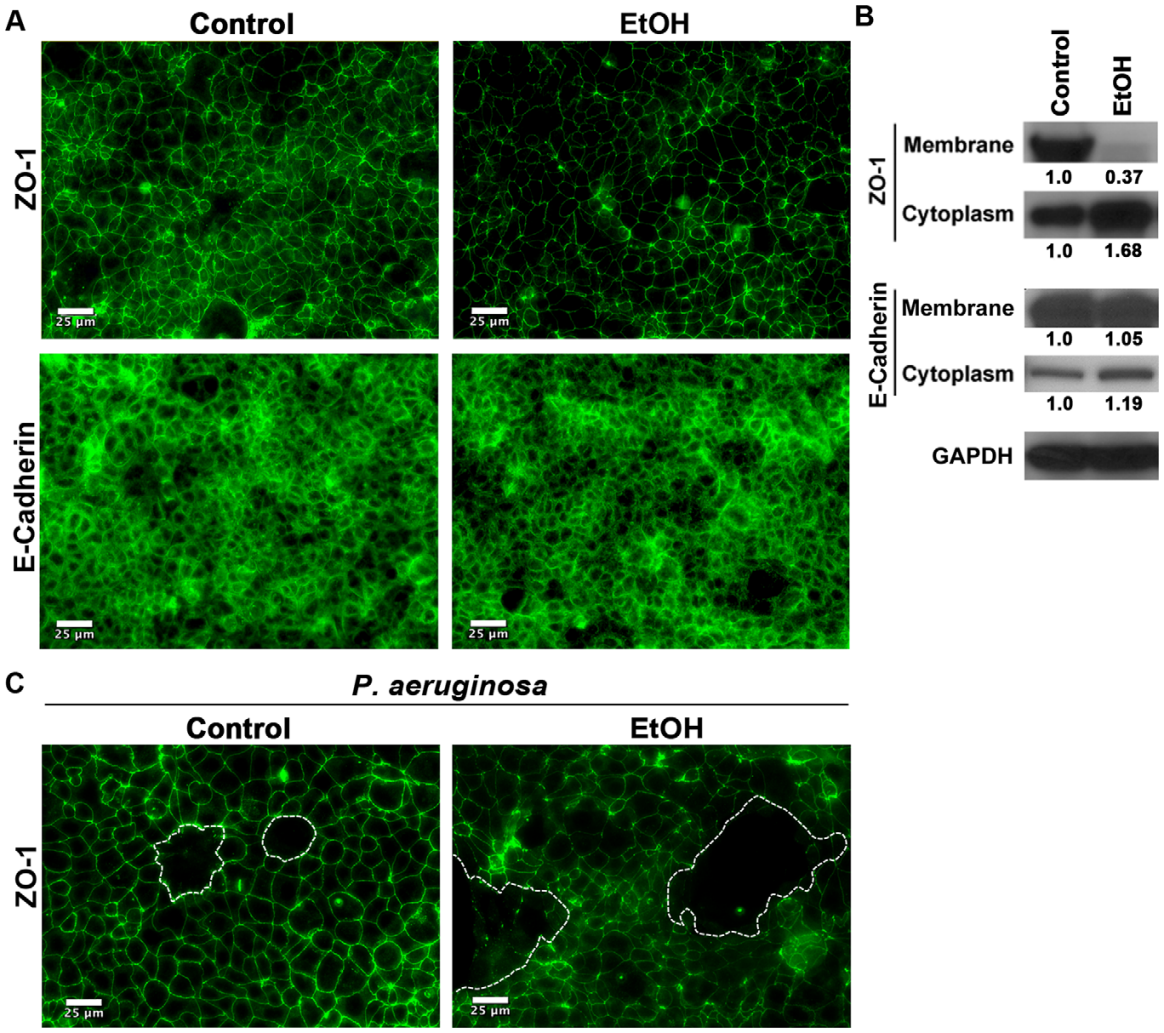
Long-term alcohol exposure adversely affects tight junction but not adherens junction. Ethanol or H_2_O-treated Caco-2 cell monolayers were fixed and the impact of alcohol treatment (EtOH) on tight and adherens juntional proteins (ZO-1 and E-cadherin respectively) was assessed by IF microscopy (**A** and **C**) or by Western blot analyses of the cytoplasmic or the membrane fractions (**B**). The ZO-1 protein levels in different fractions in (B) were determined by densitometry, normalized to GAPDH and plotted with respect to control. The data indicate that long-term alcohol exposure reduces subcellular localization of ZO-1 to tight junction structures at the membrane but it does not effect its overall expression. It also has no appreciable effect on the expression level or the subcellular localization of E-cadherin. (**C**) Ethanol or H_2_O-treated Caco-2 monolayers were infected with PAK (*P. aeruginosa*) for 5 hrs. Cells were then fixed and the impact of *P. aeruginosa* on Caco-2 cell-cell monolayer integrity was assessed by ZO-1 staining. Ethanol disruption of tight junctions as shown by IF microscopy of ZO-1 is associated with enhanced damage (white outline) due to infection. (The scale bar  = 25 µm).

Acute exposure to 0.2% (v/v) or higher ethanol concentrations has been shown to adversely impact cell-cell connections by reducing the tight and adherens junctions in epithelia [1], [4], [5], [6], [7], [8], [9]. To look at the impact of long-term ethanol exposure on epithelial junctions, we performed immunofluorescent microscopy (IF-M) on control and EtOH- treated Caco-2 cells for the tight junction protein ZO-1 and the adherens junction protein E-cadherin. ZO-1 staining at the cell-cell junctions was greatly reduced in the ethanol treated cells, compared to the control (Fig. 2A). In contrast, the adherens junction protein E-cadherin showed no difference in expression by IF-M between mock and ethanol treated cells (Fig. 2C). To determine the protein amounts and the subcellular localization of these proteins, the total membrane and cytoplasmic fractions were separated and probed for either ZO-1 or E-cadherin by Western blotting. The Western blot analyses indicated that relative to control, the ethanol treated cells had a greatly reduced level of ZO-1 in the membrane fraction. Enhanced levels in the cytoplasmic fraction indicated that alcohol exposure disrupted ZO-1 localization at the membrane, not its expression *per se* (Fig. 2B). Consistent with the IF-M expression, E-cadherin protein levels and its subcellular localization showed no difference between the control and ethanol treated Caco-2 cells (Fig. 2B). Collectively, the data indicated that like acute alcohol exposure, long-term alcohol exposure also adversely affected the tight junction structures. However and in contrast to acute exposure, long-term ethanol exposure did not affect the adherens junction.

We then asked if the reduction in the tight junction proteins at the membrane interfered with the ability of alcohol-treated Caco-2 cells to maintain barrier integrity in the face of a bacterial challenge. To this end, we conducted a short-term infection study, in which the control and the alcohol-treated confluent monolayer cells were infected for 5h with PAK, a *Pseudomonas aeruginosa* strain (Table S1). We then assessed the impact of alcohol on cell-cell connections by staining for ZO-1. *P. aeruginosa* disrupted cell-cell connections and caused substantially more damage to the alcohol-treated cells, as indicated by much larger denuded areas (outlined in white) and disorganized ZO-1 staining in the membrane (Fig. 2E). The presence of 0.2% (v/v) alcohol in the culture media did not have any effect on bacterial growth (data not shown). These data indicated that reduced tight junction fortification at the cell-cell interface may render alcoholic cells more vulnerable to bacterial pathogen- induced damage.

### Long-term Ethanol Exposure Renders Caco-2 Epithelium More Vulnerable to Bacteria-induced Wound Damage (Wound Model)

**Figure 3:**
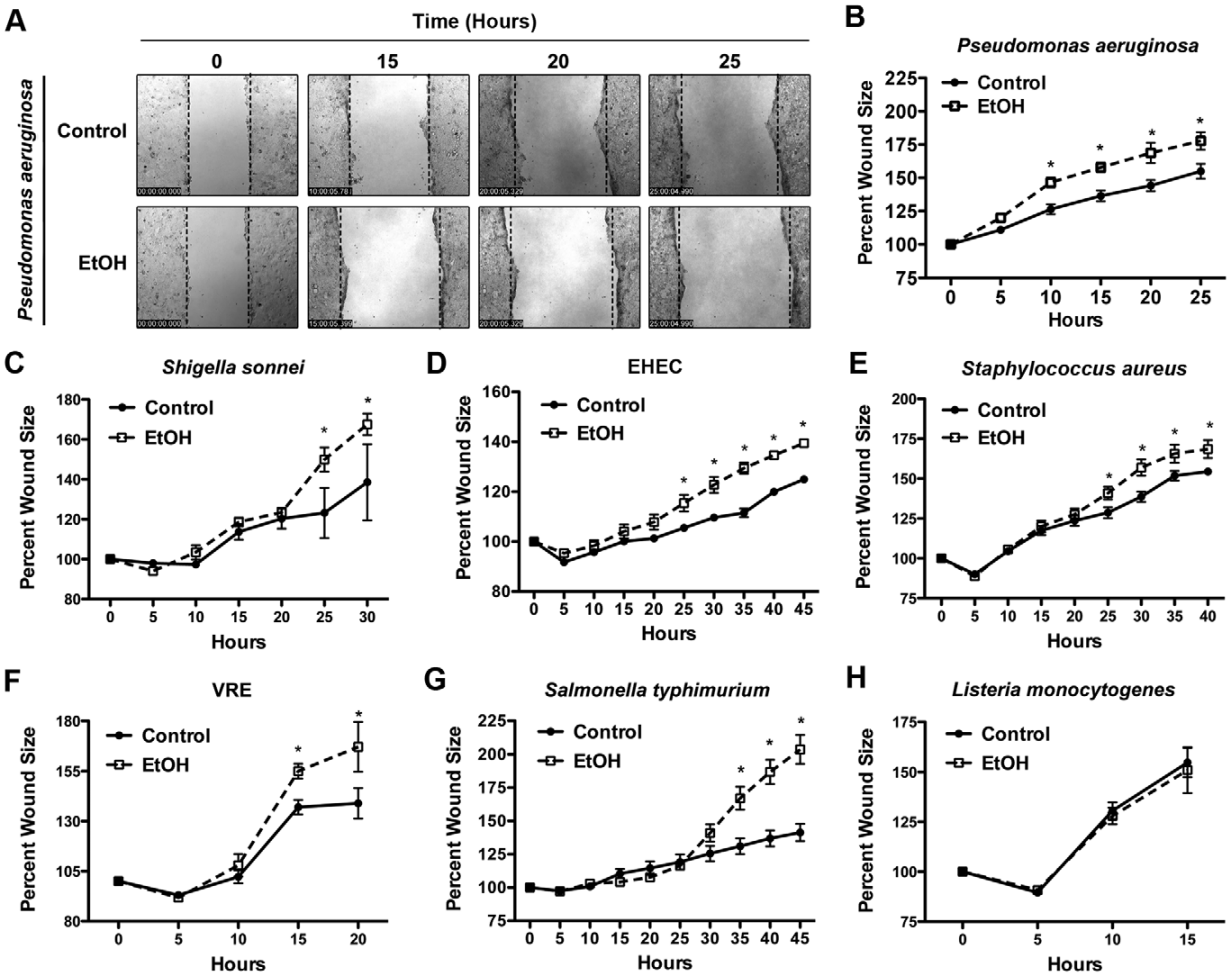
Long-term alcohol exposure renders Caco-2 wounds more vulnerable to bacteria-induced damage. (**A–H**) Caco-2 cell monolayers were scrape wounded and infected with indicated bacteria (MOI = 5). Time-lapse videomicroscopy was used to assess healing. (**A**) Representative frames of the *P. aeruginosa*-infected wounds are shown. Wound areas were measured relative to the original wound size and the tabulated results are shown in (**B**). For simplicity, the wound images for other pathogens have been omitted and only the tabulated data are shown in (**C–H**). As indicated, long-term alcohol exposure renders wounds more susceptible to damage induced by all the pathogens except *Listeria monocytogenes* (**H**). (* Indicates significance, *p*<0.05, n = 3 for each bacteria, magnification is 100×).

Epithelium is the first line of defense in the innate immune system [25]. Injured epithelium provides an opportunity for pathogens to colonize and is a preferred niche for many bacterial pathogens [26], [27], [28]. In fact, pathogens have evolved multiple virulence strategies to prevent wound healing [23], [24], [29]. We assessed the impact of alcohol exposure on the ability of epithelial cells to withstand bacterial damage in the context of wound infection using time-lapse videomicroscopy and a scratch wound model as described [21], [22], [24]. In the absence of bacterial infection, alcohol-treated Caco-2 cells healed significantly faster compared to mock cells (Figs. 1B and 1C and Movie S1). However, the situation was reversed when bacteria were present. The results indicated that when infected with *Pseudomonas aeruginosa*, *Shigella sonnei*, *Enterohaemorrhagic Escherichia coli* (EHEC), *Staphylococcus aureus,* Vancomycin-resistant *Escherichia coli* (VRE), and *Salmonella enterica* serovar *typhimurium* (*S. typhimurium*), ethanol-treated wounds (EtOH) were significantly more vulnerable to bacteria-induced damage (Figs. 3A–3F, Fig. S1, Movie S3, and data not shown. n = 3, *p*<0.05). There was one exception, *Listeria monocytogenes* showed no difference in its ability to cause wound damage in alcohol- or mock-treated cells (Fig. 3H and Movie S4). Collectively, the data indicated that alcoholic wounds were generally more susceptible to bacteria-induced wound damage.

### Long-term Ethanol Exposure Alters Caco-2 Cells Vulnerability to Bacteria-induced Cytotoxicity (Uninjured/Intact Model)

Many microbial pathogens employ a variety of strategies to induce cell death in their host as a means to establish and spread their infection [23], [30], [31], [32]. The study of pathogen-induced host cell death has gained significant momentum in recent years with the recognition that this phenomenon is not an incidental finding during infection but rather a regulated process with significant implications for pathogenesis [23], [31], [32], [33], [34].

**Figure 4:**
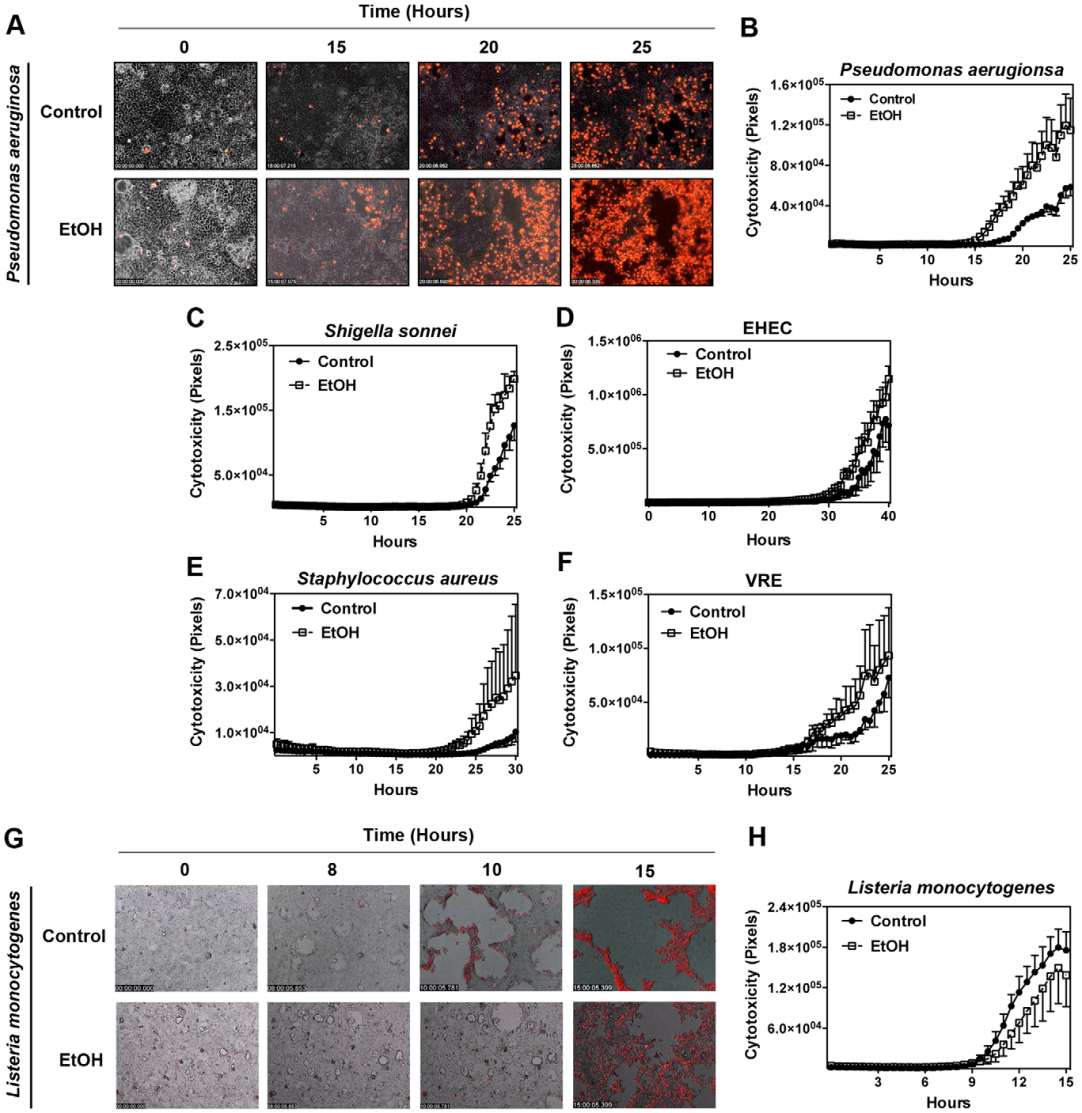
Long-term ethanol exposure alters Caco-2 cells vulnerability to bacteria-induced cytotoxicity (Uninjured/intact model). Ethanol- or mock- treated Caco-2 cells were grown to confluence. They were then infected with indicated bacterial pathogens at MOI = 5 (C–J). Cytotoxicity was assessed by the uptake of PI impermeant nuclear dye (red = dead) using time-lapse IF videomicroscopy (A and G). Cytotoxicity was assessed by measuring the total PI fluorescence at 15 min intervals by Image J and plotted in (B–F and H). Ethanol treated Caco-2 monolayers show increased cytotoxicity in the presence of *P. aeruginosa* (A and B) and other bacteria (C–F). The ethanol-treated cells are more resistant to cytotoxicity induced by *L. monocytogenes* (G and H). All frames are representative of three experiments. Infections were done at MOI = 5.

We wondered if long-term alcohol exposure rendered Caco-2 cells more vulnerable to pathogen-induced cytotoxicity. To this end, we infected the ethanol and the mock-treated Caco-2 confluent monolayers with the aforementioned pathogenic bacteria listed in the Table S1. We measured cytotoxicity by the uptake of propidium iodide (PI) impermeant nuclear dye, which fluoresces red in dead or dying cells, at 15 min increments using IF time-lapse videomicroscopy, as described in materials and methods and [23]. In the absence of bacteria, alcohol-treated Caco-2 cells exhibited slightly higher levels of cytotoxicity as compared to the mock (Figs. 1D and 1E and Movie S2). In the presence of bacteria however, alcohol-treated cells were generally more susceptible to pathogen-induced cytotoxicity. Alcohol-treated Caco-2 cells infected with *P. aeruginosa, S. sonnei*, EHEC, *S. aureus,* and VRE, succumbed to cytotoxicity earlier and to a higher extent than did the control cells (Figs. 4A–4F, Fig. S2, Movies S5 and S6, and data not shown). Again, the exception was *L. monocytogenes* where alcohol-treated Caco-2 cells were more resistant to cytotoxicity induced by this pathogen (Fig. 4G–4H and Movie S7).

### The Adverse Effects of Long-term Alcohol Exposure on Caco-2 Cells are Partially Reversible

**Figure 5:**
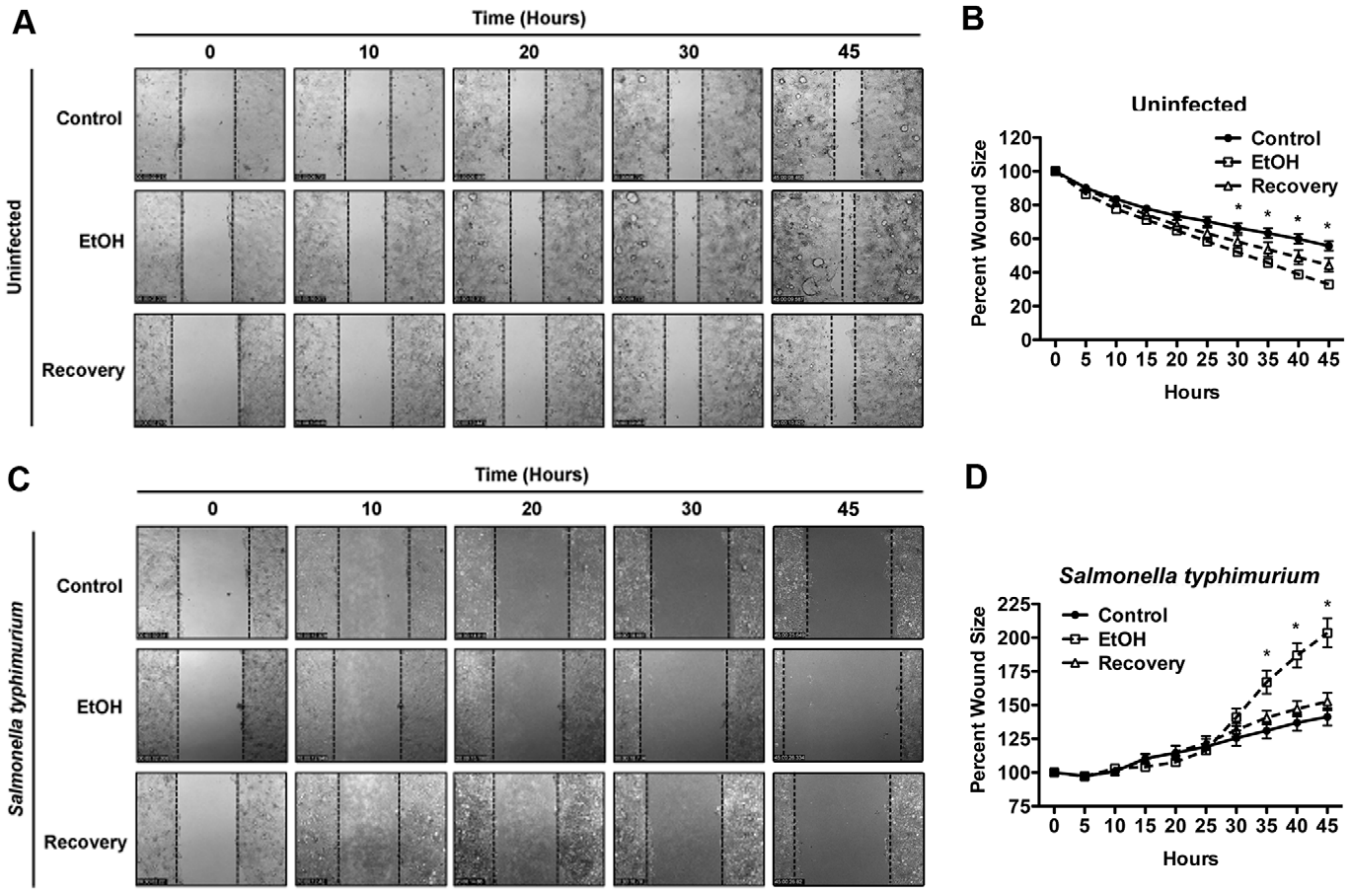
Stopping alcohol exposure can partially reverse the negative impacts of long-term alcohol exposure. Alcohol-treated cells were allowed to recover in the absence of ethanol for 2 weeks (Recovery). EtOH, control, and Recovery Caco-2 confluent monolayers were scrape-wounded and their healing capacity in the absence (**A–B**) or presence of *Salmonella typhimurium* at MOI = 10 (**B–D**), was assessed by time-lapse videomicroscopy. Representative movie frames at indicated time points are shown for the uninfected cells in (**A**) and for the infected cells in (**C**), and the corresponding tabulated results are shown in (**B**) and (**D**) respectively. In the absence of bacteria, ethanol-treated cells heal significantly faster than the mock-treated cells (control) and the recovered cells (Recovery) show intermediate wound closure (**A–B**). In the presence of *Salmonella typhimurium*, the recovered cells exhibit similar phenotype to mock-treated cells in that they are significantly more resistant to wound damage induced by *S. typhimurium* than the EtOH cells (* indicates significance relative to EtOH cells, *p*<0.05, n = 3).

**Figure 6:**
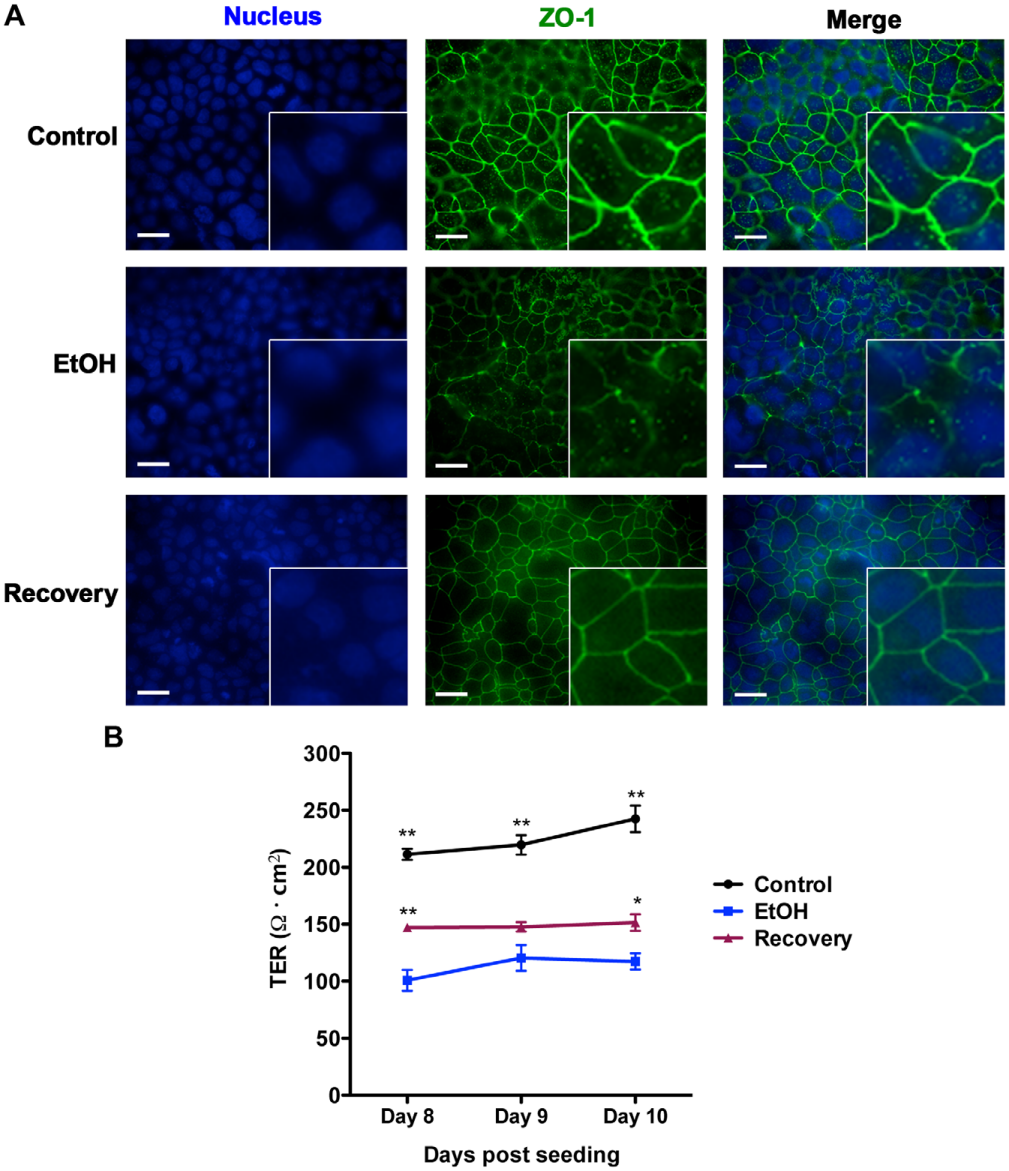
Stopping alcohol exposure can partially reverse the negative impacts of long-term alcohol exposure on junctional structure and epithelial barrier function. Alcohol-treated cells were allowed to recover in the absence of ethanol for 2 weeks (Recovery). (A) EtOH, Control, and Recovery Caco-2 cell monolayers were fixed and the impact of alcohol treatment (EtOH) and its stoppage (Recovery) on tight junctional protein ZO-1 membrane localization was assessed by IF microscopy and representative images are shown. Data indicate that in the recovery cells, ZO-1 membrane localization shows intermediate phenotype in that it is more organized than in the EtOH-treated cells, although, ZO-1 staining at the membrane (as depicted by fluorescence intensity in the green panel) is reduced compared to control cells. (Scale bar = 25 µm. Inserts are representative field of cells in each image, magnified 3x). (B) EtOH, Control, and Recovery Caco-2 cells were cultured until confluent. The impact of alcohol treatment (EtOH) and its stoppage (Recovery) on epithelial barrier function was assessed by TER measurements on indicated days post seeding. As indicated, long-term alcohol exposure significantly reduces resistance across EtOH monolayer while Recovery cell monolayers exhibit intermediate resistance profile. (* and ** indicate significance with *p*<0.05 and *p*<0.001 respectively. N = 4).

We then asked whether the adverse effects of long-term alcohol exposure could be reversed upon stoppage of ethanol treatment. To this end, we cultured the long-term ethanol-treated Caco-2 cells in alcohol-free media for two additional weeks (Recovery phase). We then assessed the impact of alcohol treatment stoppage on the ability of ethanol-treated Caco-2 cells to heal in the absence or presence of *S. typhimurium*. In the absence of bacteria, alcohol-treated Caco-2 cells after recovery phase (Recovery) healed faster than the control but slower that the long-term alcohol-treated cells, exhibiting an intermediate phenotype (Figs. 5A and 5B, and Movie S8). When infected with *S. typhimurium*, the recovery cells were significantly more resistant to *S. typhimurium*- induced wound damage than the long-term alcoholic cells (EtOH) but appeared to be less resistant than the control group, although the differences between the recovery and the control groups did not reach statistical significance (Figs. 5C and 5D and Movie S9, *p*<0.05 between EtOH and Recovery groups vs. control). Furthermore, recovery cells also exhibited intermediate phenotypes with respect to ZO-1 subcellular localization to tight junction structures (Fig. 6A) and with respect to paracellular epithelial permeability, as assessed by TER (transepithelial electrical resistance) analyses (Fig. 6B, *p*<0.05 between Recovery and EtOH and *p*<0.001 between Control and EtOH, n = 8).

## Discussion

We established a simple model system to study how the long-term alcohol exposure may influence the epithelial barrier’s capacity to maintain its integrity when confronted with pathogenic bacteria under the intact (uninjured) or wound conditions. In this communication, we report that daily exposure to 0.2% (v/v) ethanol for 2 months weakens the tight junction structure and renders Caco-2 cells substantially more vulnerable to cellular damage caused by most bacterial pathogens under both the intact and the wound conditions (Figs. 2, 3, 4, 5, S1 and S2, and Movies S3, S5, S6, and S9).

Our data indicate that long-term alcohol exposure reduces the ability of the gut epithelium to maintain barrier integrity due to its enhanced sensitivity to pathogen-induced cytotoxicity and cellular damage, thus enabling bacterial and other toxins that normally cannot pass through the mucosa to enter the systemic circulation more readily. Consistent with this notion, mucosal damage in the small intestine with a loss of epithelium, hemorrhagic erosions, and hemorrhage in the lamina propria were reported in rodents or dogs subjected to alcohol [35]. The alcohol-induced mucosal damage was shown to correlate with bacterial outgrowth [36]. Nearly 50% of the alcoholics were found to have increases in the total number of bacteria in the jejunal and duodenal juices in small intestine [36]. Interestingly, reducing microbial flora by oral administration of broad spectrum antibiotics (polymyxin B and neomycin) significantly reduced the endotoxin level in the blood and attenuated alcohol-induced liver damage in rats following long-term exposure to ethanol, as measured by serum transaminase levels and the pathology score [37]. Combined, these data strongly suggest that the live bacterial pathogens may be to blame for epithelial barrier disruption, the enhanced permeability to bacterial products and bacterial infection in long-term alcoholics, and that the damage to the epithelial barrier may benefit bacteria in that environment.

*Listeria monocytogenes* was the lone exception in these studies. Our data demonstrate that long-term exposure to ethanol renders Caco-2 confluent monolayers more resistant to *L. monocytogenes* than mock-treated cells, particularly in the intact monolayer condition (Figs. 3H and 4H, and Movies S4 and S7). This finding may not be surprising given that *L. monocytogenes* is an intracellular pathogen that requires close contacts between neighboring epithelial cells as it spreads from cell to cell via an actin-based motility apparatus [38] and considering how alcohol exposure loosens cell-cell connections through its adverse impact on tight junctional structures (Figs. 2 and 6).

These data also suggest that long-term alcohol exposure may be an important factor in shaping the microflora because it can exert a selective pressure on bacteria. Consistent with this notion, we previously demonstrated that long-term alcohol consumption alters colonic mucosa-associated bacterial microbiota composition, culminating in dysbiosis in rats [39]. A shift toward pathogenic bacteria would certainly have important implications for the pathology associated with alcohol consumption as commensal microflora serve many protective functions against pathogenic bacteria (reviewed in [40]).

Interestingly, in the absence of bacteria, long-term alcohol treated Caco-2 cells exhibited significantly faster healing capacity (Figs. 1B and 1C and Movie S1). Enhanced cell migration in alcohol treated cells may be due to alcohol’s stimulatory effect to promote epithelial-mesenchymal-transition (EMT) transition [41]. During the start phase of normal wound healing, epithelial cells at the wound edges undergo EMT becoming more like fibroblast cells [42], [43], [44]. EMT transition facilitates and enhances their migrating capacity into denuded areas [42], [43], [44]. As leading wound edges come close together, these cells slow down and reverse course, undergoing the mesenchymal-epithelial-transition (MET), in order to regain their epithelial characters so as to re-differentiate and re-establish connections in a process known as re-epithelization [43], [44]. Our data suggest that long-term alcohol exposure interferes with the mesenchymal-epithelial-transition, as alcoholic cells continue to maintain fast migration kinetics during the stop phase of wound healing [42].

Encouragingly, alcohol-treated cells partially regain their ability to remedy the negative impact of alcohol on junctional structures and epithelial permeability and to withstand infection and correct their migration dynamics in the absence of bacteria, when ethanol treatment was ceased for two weeks (Figs. 5 and 6 and Movies S8 and S9), indicating that alcohol’s deleterious effects on the cells may be at least partially reversible.

## Materials and Methods

### Cell Culture

Cells were seeded in 24- or 6- well dishes and grown in DMEM (Gibco) media supplemented with 10% FCS, 1% L-glutamine, and 1% penicillin and streptomycin. Adherent Caco-2 cells were either given media containing 0.2% ethanol (EtOH) or media containing 0.2% H_2_O (control) for at least 2 months prior to experiments. Cells were passaged approximately every 3 days when they reach about 80% confluency. Recovery cells were ethanol treated for 2 months as described above and then grown without ethanol for at least 2 weeks. In all experiments, 20% more EtOH cells were seeded to account for reduced proliferative capacity in EtOH-treated cells compared to control Caco-2 cells. Experiments were performed when cells were fully confluent. At the time of experiments, cell counts were performed to ensure similar numbers.

### Reagents

Purchased reagents and kits include the following: BCA kit (Pierce); ECL reagent (Pierce); VectaShield mounting medium with DAPI (Vector Labatories); Hyperfilm ECL (GE). All chemicals were from Sigma unless otherwise stated. Antibodies were ZO-1 (#610966, BD Transduction) and E-cadherin (#3195, Cell Signaling). Horseradish peroxidase-conjugated antibodies were obtained from Cell Signaling.

### Western Blot Fractionation

Cells were grown until confluent on 100 cm^2^ dishes. Cells were washed with PBS and spun at 500×*g* to pellet cells. The pellet was resuspended in a homogenization buffer containing 250 mM sucrose, 40 mM Tris/HCl, pH 7.5, 10 mM MgCl_2_ 2 mM CaCl_2_ 1 mM phenylmethylsulfonyl fluoride, 1 mM sodium orthovanadate and CompleteMini protease inhibitor cocktail (Roche) and homogenized in a 2 ml Dounce homogenizer with 70 downward strokes. Lysates were spun at 800×*g* to pellet the nuclei. The supernatant was then ultracentrifuged at 125,000×*g* for 1 h. The supernatant containing the cytoplasm was carefully removed from the membrane pellet. The membrane pellet was resuspended in 200 µl lysis buffer (PBS with 1% Triton-X100 and protease inhibitor). Protein content was analyzed by BCA analysis (Pierce). Samples were prepared and tested by Western blot as previously described [21], [24]. Briefly, samples were resolved by 10% SDS-PAGE, transferred to a PVDF membrane, blocked with 5%milk and probed overnight with antibody. Blots were then probed with a horseradish peroxidase-conjugated antibody and developed with ECL.

### Wound Healing Assays (Wound model) (n = 3)

Caco-2 cells were grown into a confluent monolayer on 24 or 48-well plates. Monolayers were scrape wounded with a plastic pipette tip as described [21], [24]. Cells were washed three times with MEM Eagle’s to remove serum and antibiotics and a linear wound was made with a plastic pipette tip. After wounding, the cells were washed 3 times to remove the cell debris and grown in 1 ml media with or without antibiotics. For infections, bacteria were grown overnight. On the day of experiment, bacteria were diluted with MEM Eagle’s plus 10% FBS and the MOI determined by titering the dilution; an inoculum of 100 µL of bacteria per well (24 well plate) at an OD_600_ of 0.05 corresponded to an MOI of approximately 5. Time-lapse videomicroscopy was performed as described [21], [24] using an AxioVert Z1 microscope (Zeiss) and AxioVision v4.2 software. Wells were observed at 100× magnification using DIC optics. The wound sizes ware measured by determining the surface areas of the denuded regions using NIH image J software which outlines wound edges and calculates the area.

### Cytotoxicity Assays (Monolayer Intact Model) (n = 3)

Caco-2 cells were grown in a 24-well plate until confluent. Cells were washed with pre-warmed PBS and grown in media with or without antibiotics. Propidium iodide (PI) was added at 7 µg/ml final concentration and bacteria were prepared and added as above. Immunofluorescent (IF) Time-lapse videomicroscopy was performed using 200× magnification and phase-contrast optics. Cytotoxicity was determined from each frame, at 15 min interval, using the PI channel and analyzed in Image J v1.46 by setting an appropriate threshold to isolate PI-positive cells and image stacks were then analyzed for total positive pixels per frame.

### Immunofluorescent Static Microscopy

Cells were grown as above on coverslips treated with poly-l-lysine and human fibronectin (40 µg/ml) (Millipore). Confluent cells were either left uninfected or infected with PAK (Pseudomonas aeruginosa) for 5 h. Coverslips were fixed with 10% TCA, permeabilized with PBS and glycine (30 mM) and 1% Triton X-100, and blocked with PBS and glycine with 3% FCS. Primary antibodies were added 1∶50 overnight in blocking buffer. Following washing, fluorochrome-conjugated secondary antibodies were added 1∶300. Coverslips were mounted on slides with DAPI media and sealed with clear nail polish. Fluorescent microscopy was performed with the same microscope as above.

### Epithelial Barrier Function Analysis (n = 4)

Transepithelial Electrical Resistance (TER) studies were performed as described [45], [46]. Briefly, cells were seeded in 200 µl of either regular medium or medium containing 0.2% ethanol, as described above, in Transwell plates (Fisher Scientific, CAT#3470). 1 ml of same media was added to the lower chamber. Cells were incubated at 37°C, 5% CO_2_. Adherent cells receive fresh media containing either 0.2% ethanol or 0.2% H_2_O. When cells were confluent (around day 8 day post seeding), TER measurements were taken with World Precision Instruments (serial# 850936076).

### Statistics

All studies were done at least in triplicates or as indicated. Statistical significance was determined by 2-way ANOVA and Bonferroni post-test using Prism 5.0a (Graphpad). P values <0.05 were defined as significant.

## Supporting Information

Figure S1 **Long-term alcohol exposure renders Caco-2 cell wounds more vulnerable to EHEC-induced damage.** Mock and ethanol treated Caco-2 monolayers were scrape-wounded and infected with EHEC at MOI = 5. The impact of alcohol on the ability of Caco-2 cells to withstand wound damage induced by EHC was assessed by time-lapse videomicroscopy. Representative frames from 3 experiments, taken at indicated time points, indicate that long-term alcohol exposure renders the wounds more susceptible to EHEC-induced damage. The data from these studies were plotted in Fig 3D.
(TIF)

Figure S2 **Long-term alcohol exposure leads to enhanced cytotoxicity induced by** ***S. aureus*** **in a confluent monolayer.** Mock and ethanol treated confluent monolayers were infected with *S. aureus* (Newman strain) at MOI = 5 in the presence of PI and *S. aureus*- induced cytotoxicity was assessed at 15-min intervals by time-lapse IF time-lapse videomicroscopy. The data were tabulated and shown in Fig 4E. Representative frames from 3 experiments, taken at indicated time points, indicate that ethanol treated monolayers are more vulnerable to the *S. aureus*- induced cytotoxicity (red = dead or dying) at earlier time points compared to mock treated cells.
(TIF)

Table S1 **Bacterial strains used in this study.**
(DOCX)

Movie S1 **Long-term alcohol exposure leads to faster wound repair.** Ethanol (EtOH) and mock treated (Control) Caco-2 cell monolayers were scrape-wounded and their capacity to heal was assessed by time-lapse videomicroscopy. Images were captured every 15 min at 200× magnification and are shown at 15 fps. Data indicate that EtOH monolayers heal significantly faster the control monolayer.
(MOV)

Movie S2 **Long-term alcohol exposure does not lead to enhanced cytotoxicity in uninfected Caco-2 cell monolayers.** Confluent monolayers of control and ethanol EtOH Caco-2 cells were observed by time-lapse IF video microscopy. Cytotoxicity was assessed by the uptake of the impermeant nuclear fluorescent dye PI (red = dead). Images were captured every 15 min at 200× magnification and shown at 15 fps.
(MOV)

Movie S3 **Long-term alcohol exposure renders wounds more susceptible to** ***Pseudomonas aeruginosa*** **- induced wound damage.** Control) and EtOH cell monolayers were scrape-wounded and infected with PAK (*P. aeruginosa*) (MOI = 5). Wound susceptibility to bacteria-induced damage was assessed by time-lapse video microscopy. Images were captured every 15 min at 200× magnification and are shown at 15 fps.
(MOV)

Movie S4 **Long-term alcohol exposure does not affect** ***Listeria monocytogenes*** **- induced wound damage.** Mock and ethanol treated cell Caco-2 cell monolayers were scrape-wounded and infected with *L. monocytogenes* (EGD strain) (MOI = 5). Wound susceptibility to bacteria-induced damage was assessed by time-lapse video microscopy. Images were captured every 15 min at 200× magnification and shown at 15 fps.
(MOV)

Movie S5 **Long-term alcohol exposure renders Caco-2 cell monolayers more susceptible cytotoxicity induced by** ***Pseudomonas aeruginosa*** **.** Confluent monolayers of mock and ethanol treated Caco-2 cells were infected with PAK (MOI = 5). Cytotoxicity was assessed by the uptake of the impermeant nuclear fluorescent dye PI (red = dead). Images were captured every 15 min at 200× magnification and shown at 15 fps.
(MOV)

Movie S6 **Long-term alcohol exposure renders Caco-2 cell monolayers more susceptible to cytotoxicity induced by** ***Staphylococcus aureus*** **.** Confluent monolayers of mock and ethanol treated Caco-2 cells were infected with *S. aureus* (Newman strain) (MOI = 5). Cytotoxicity was assessed by the uptake of the impermeant nuclear fluorescent dye PI (red = dead). Images were captured every 15 min at 200× magnification and shown at 15 fps.
(MOV)

Movie S7 **Ethanol treated Caco-2 cell monolayers are less susceptible to cytotoxicity induced by** ***Listeria monocytogenes*** **.** Confluent monolayers of mock and ethanol treated Caco-2 cells were infected with *L. monocytogenes* (EGD) (MOI = 5). Cytotoxicity was assessed by the uptake of the impermeant nuclear fluorescent dye PI (red = dead). Images were captured every 15 min at 200× magnification and shown at 15 fps.
(MOV)

Movie S8 **Stopping ethanol treatment for 2 weeks partially restores wound healing kinetics in the long-term ethanol treated Caco-2 wounded epithelium.** Recovery cells were generated by growing long-term ethanol-treated Caco-2 cells in ethanol-free media for 2 weeks. Monolayers of control, EtOH, and recovery cells were scrape-wounded and their ability to heal was assessed by time-lapse videomicroscopy. Images were captured every 15 min at 200× magnification and shown at 15 fps.
(MOV)

Movie S9 **Stopping ethanol treatment for 2 weeks restores the ability in the Caco-2 wounded epithelium to withstand further bacteria-induced damage.** Recovery cells were generated by growing long-term ethanol-treated Caco-2 cells in ethanol-free media for 2 weeks. Monolayers of control, EtOH, and recovery cells were scrape-wounded and infected with *S. typhimurium* (14028 s strain) (MOI = 5). Wound susceptibility to *S. typhimurium*-induced damage was assessed by time-lapse video microscopy. Images were captured every 15 min at 200× magnification and shown at 15 fps.
(MOV)
