# Supplementary figures and images for: Chronic Alcohol Exposure Renders Epithelial Cells Vulnerable to Bacterial Infection
Source: PLoS One. 2013 Jan 24;8(1):e54646. doi: 10.1371/journal.pone.0054646 (PMC3554638; doi:10.1371/journal.pone.0054646)

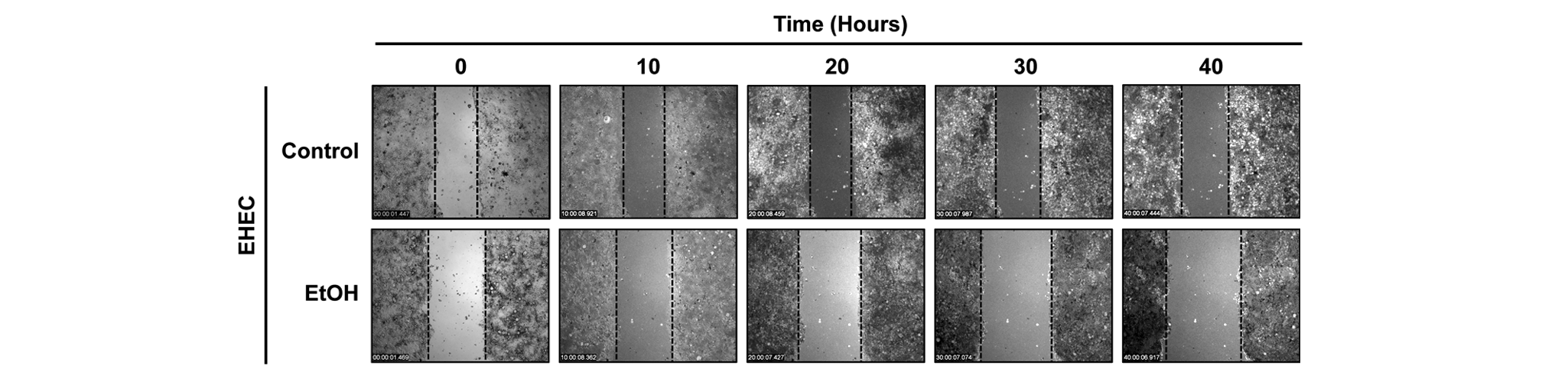

Supplement: Figure S1 — Long-term alcohol exposure renders Caco-2 cell wounds more vulnerable to EHEC-induced damage. Mock and ethanol treated Caco-2 monolayers were scrape-wounded and infected with EHEC at MOI = 5. The impact of alcohol on the ability of Caco-2 cells to withstand wound damage induced by EHC was assessed by time-lapse videomicroscopy. Representative frames from 3 experiments, taken at indicated time points, indicate that long-term alcohol exposure renders the wounds more susceptible to EHEC-induced damage. The data from these studies were plotted in Fig 3D. (TIF) [file pone.0054646.s001.tif]

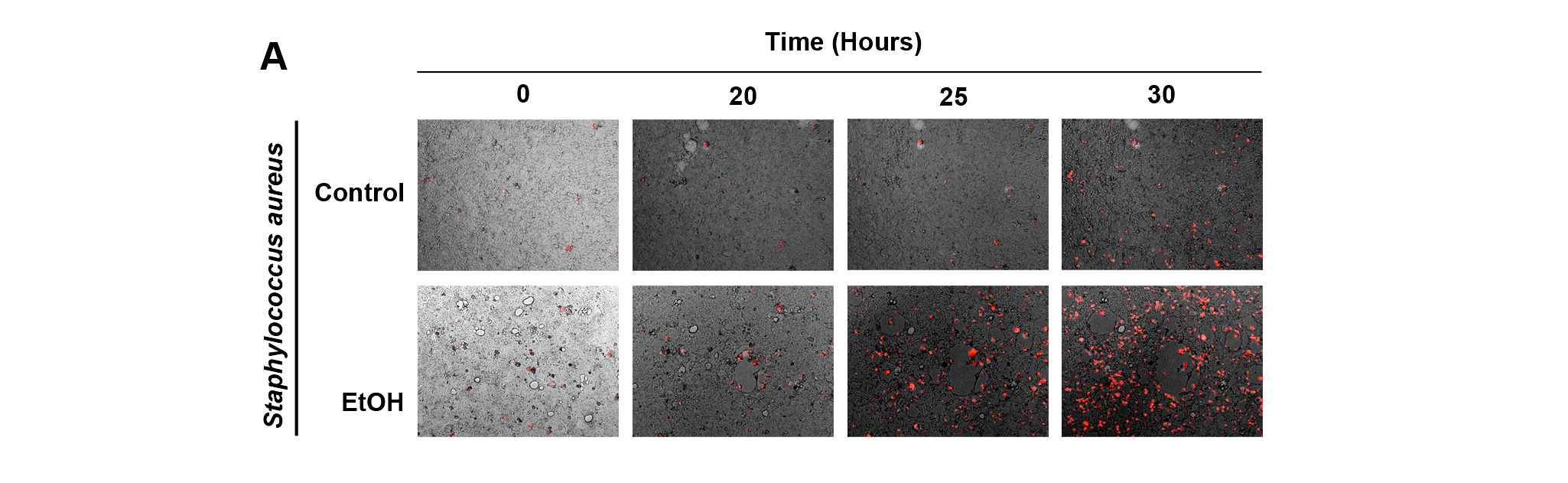

Supplement: Figure S2 — Long-term alcohol exposure leads to enhanced cytotoxicity induced by S. aureus in a confluent monolayer. Mock and ethanol treated confluent monolayers were infected with S. aureus (Newman strain) at MOI = 5 in the presence of PI and S. aureus- induced cytotoxicity was assessed at 15-min intervals by time-lapse IF time-lapse videomicroscopy. The data were tabulated and shown in Fig 4E. Representative frames from 3 experiments, taken at indicated time points, indicate that ethanol treated monolayers are more vulnerable to the S. aureus- induced cytotoxicity (red = dead or dying) at earlier time points compared to mock treated cells. (TIF) [file pone.0054646.s002.tif]
