# Supplementary material for: Chronic Alcohol Exposure Renders Epithelial Cells Vulnerable to Bacterial Infection
Source: PLoS One. 2013 Jan 24;8(1):e54646. doi: 10.1371/journal.pone.0054646 (PMC3554638; doi:10.1371/journal.pone.0054646)
Supplement: Table S1 — Bacterial strains used in this study. (DOCX) [file pone.0054646.s003.docx]

**Table S1.** Bacterial strains used in this study.

| **Bacteria** | **Strain** | **Source** |
| --- | --- | --- |
| *Pseudomonas aeruginosa* | PAK | [[1](#_ENREF_1)] |
| *Salmonella enterica serovar Typhimurium* | 14028s | Dr. Linda Kenny [[2](#_ENREF_2)] |
| *Staphylococcus aureus* | Newman | Dr. Taeok Bae [[3](#_ENREF_3)] |
| Vancomycin resistant *Escherichia coli* | Clinical isolate | Dr. Kamal Singh |
| Enterohemorrhagic *Escherichia coli* | O157:H7 | Dr. Gail Hecht [[4](#_ENREF_4)][[4](#_ENREF_4)] |
| *Shigella sonnei* | Clinical isolate | Dr. Kamal Singh |
| *Listeria monocytogenes* | EGD | Dr. Nancy Freitag [[5](#_ENREF_5)] |

1. Kierbel A, Gassam A, Mostov K, Engel J (2005) The Phosphoinositol-3-kinase-Protein kinase B/Akt pathway is critical for Pseudomonas aeruginosa strain PAK internalization. Mol Biol Cell 16: 2577.

2. Feng X, Oropeza R, Kenney LJ (2003) Dual regulation by phospho-OmpR of ssrA/B gene expression in Salmonella pathogenicity island 2. Mol Microbiol 48: 1131-1143.

3. Duthie ES, Lorenz LL (1952) Staphylococcal coagulase; mode of action and antigenicity. Journal of general microbiology 6: 95-107.

4. Hecht G, Marrero JA, Danilkovich A, Matkowskyj KA, Savkovic SD, et al. (1999) Pathogenic *Escherichia coli* increase Cl- secretion from intestinal epithelia by upregulating galanin-1 receptor expression. J Clin Invest 104: 253-262.

5. Miner MD, Port GC, Freitag NE (2008) Functional impact of mutational activation on the Listeria monocytogenes central virulence regulator PrfA. Microbiology 154: 3579-3589.
